# LAIV Mutations Selectively Alter Influenza Viral RNA Polymerase Function, Favoring Transcription over Genome Synthesis

**DOI:** 10.3390/v17111412

**Published:** 2025-10-23

**Authors:** Justin R. Leach, Adrian Oo, Aitor Nogales, Sebastian I. Bosch, Luis Martínez-Sobrido, Changyong Feng, Baek Kim, Stephen Dewhurst

**Affiliations:** 1 Department of Microbiology and Immunology, University of Rochester School of Medicine and Dentistry, Rochester, NY 14642, USA; justin_leach@urmc.rochester.edu (J.R.L.);; 2 Department of Pediatrics, Emory University School of Medicine, Atlanta, GA 30322, USA; adrianoo@nus.edu.sg (A.O.); baek.kim@emory.edu (B.K.); 3 Animal Health Research Centre, Centro Nacional Instituto de Investigación y Tecnología Agraria y Alimentaria, 28040 Madrid, Spain; 4 Texas Biomedical Research Institute, San Antonio, TX 78227, USA; 5 Department of Biostatistics and Computational Biology, University of Rochester School of Medicine and Dentistry, Rochester, NY 14642, USA; changyong_feng@urmc.rochester.edu

**Keywords:** LAIV, influenza, polymerase, vaccination, H1N1

## Abstract

Influenza viruses cause mild to severe lower respiratory infections, sometimes resulting in hospitalization and death. Vaccination remains the primary prophylactic strategy. Live attenuated influenza vaccines (LAIVs) efficiently induce antiviral immune responses and contain temperature-sensitive and cold-adapted mutations that render them safe. These mutations are principally located in the PB1 and PB2 subunits of the viral RNA polymerase, but the mechanism by which they attenuate the virus is unclear. We introduced the PB1 and PB2 mutations from two LAIV backbones, A/Ann Arbor/6/1960 H2N2 (AA) and A/Leningrad/134/17/1957 H2N2 (Len), into the model influenza strain A/Puerto Rico/8/1934 H1N1 (PR8). In contrast to the wild-type (WT) PR8 polymerase, the two “PR8-LAIV” polymerase complexes demonstrated maximal activity at cold temperatures (30–32 °C) and greatly reduced activity at elevated temperatures (>37 °C). To further understand the impact of the LAIV mutations, we infected MDCK cells with WT and mutated PR8 viruses that contain the Len and AA LAIV mutations in PB1 and PB2. The PR8-LAIV mutant viruses exhibited a selective, temperature-dependent defect in the replicase activity of the viral RNA polymerase relative to WT PR8, while also demonstrating a temperature-dependent enhancement in the transcriptional activity of the enzyme. In addition, the PR8-LAIV mutant viruses produced similar levels of viral proteins to WT PR8 at 37 °C, but greatly (2–3 log_10_) reduced levels of infectious viral progeny. Collectively, these data show that LAIV mutations selectively alter influenza viral RNA polymerase function, favoring transcription over genome synthesis at 37 °C, thereby preserving viral antigen production while also contributing to viral attenuation.

## 1. Introduction

Influenza viruses are segmented, negative-sense RNA viruses belonging to the Orthomyxoviridae family which can cause severe lower respiratory infections. In the 2023–2024 influenza season, the CDC reported an estimated 40 million symptomatic illnesses, 470,000 hospitalizations and 28,000 deaths due to influenza in the United States (U.S.) [1]. While vaccination remains the most effective form of influenza prophylaxis, influenza vaccines have been limited in their overall effectiveness, ranging from roughly 30–50% protective efficacy each year over the past 10 years—much lower than most other vaccines [2]. This is partly because influenza vaccines must be reformulated annually to protect against the viral strains predicted to be circulating in the upcoming flu season.

The trivalent Inactivated Influenza Vaccine (IIV3) is currently the most common influenza vaccine in the U.S. (for the 2024–2025 season) and efficiently elicits antiviral antibodies. Live Attenuated Influenza Viruses (LAIVs) differ from IIV3 in that they can induce both cellular and humoral immune responses from vaccinated individuals [3,4,5,6,7], due to the fact that they actively replicate in human cells [8]. This is a potentially important advantage, in that influenza strains can readily evade antibody recognition due to antigenic drift in variable domains of the viral surface proteins (hemagglutinin (HA) and neuraminidase (NA) [9]), whereas evasion of cellular immune responses directed against conserved epitopes is harder to achieve [10].

Current LAIVs were originally developed by cold adaptation of wild-type (WT) viruses, resulting in the selection of virus mutants that replicate efficiently at low temperatures (e.g., 25–30 °C) [11] but not at higher temperatures (e.g., 37–42 °C). As a result, such viruses are attenuated in humans, but still capable of eliciting robust antiviral immune responses. Genetic analyses of cold-adapted LAIVs have shown that these viruses contain multiple mutations in the genes (PB2, PB1 and PA) that encode the tripartite viral RNA dependent RNA polymerase (RdRp) [12,13,14]. Specifically, LAIVs licensed for use in the U.S. incorporate the genetic backbone from a cold-adapted mutant of the influenza A/Ann Arbor/6/1960 H2N2 virus (hereafter referred to as LAIV-AA or AA), which has 4 mutations that map in PB1 (K391E, E581G, and A661T) and PB2 (N265S) [12,14]. Similarly, LAIVs used in Russia and a number of other countries incorporate the genetic backbone from a cold-adapted mutant of the influenza A/Leningrad/17/57 H2N2 virus (hereafter referred to as LAIV-Len or Len), which has 3 mutations that map to PB1 (K265N and V591I) and PB2 (V478L), plus an additional mutation in NEP (M100I).

Previous studies by our group and others have shown that these polymerase gene mutations are necessary and sufficient to confer live-attenuated (att) and cold-adapted (ca) phenotypes on other strains of influenza A viruses, including the well-characterized mouse-adapted virus A/Puerto Rico/8/1934 H1N1 (hereafter referred to as PR8) [15]. The underlying mechanism(s) for this, however, remains unclear. In this study, we therefore introduced the PB1 and PB2 mutations from LAIV-AA and LAIV-Len into the corresponding polymerase genes of PR8 and then examined the effects of these mutations on viral RNA polymerase activity both in vitro (by conducting biochemical analyses of purified polymerase complexes) and in cultured cells (by analyzing viral RNA synthesis in infected cells).

Our in vitro studies revealed that purified PR8 polymerase complexes containing LAIV mutations (both AA and Len) are more enzymatically active at lower temperatures than the WT PR8 polymerase complex. Our in vivo studies in virally infected cells provided additional, complementary insight into the functional effects of the LAIV mutations. Specifically, we observed that the PR8-WT virus and the PR8-AA and PR8-Len mutant viruses produced very similar amounts of viral mRNA (mRNA) and viral genomic RNA (vRNA) in MDCK cells at 33 °C following infection at a multiplicity of infection (MOI) of 1. However, at 37 °C, the PR8-AA and PR8-Len viruses produced only approximately half the amount of vRNA that was generated by the WT virus, while the levels of viral mRNA that they produced were roughly 2-fold higher than the WT virus. This suggests that the LAIV mutations confer a selective, ts, defect on the replicase function of the viral RNA polymerase, while leaving the transcriptase enzyme activity of the enzyme intact (or even enhanced). Consistent with the robust levels of viral mRNA at 37 °C in MDCK cells infected by PR8-AA and PR8-Len, the production of viral proteins (NP, and NS1) in such cells was also equivalent to that in cells infected with PR8-WT. In contrast, there was a 2–3 log_10_ reduction in the production of infectious progeny virus by the PR8-AA and PR8-Len viruses in MDCK cells at 37 °C, as compared to PR8-WT. We attribute this, at least in part, to the reduced levels of genomic vRNA. Collectively, these findings suggest a mechanistic basis for the attenuated and temperature-sensitive phenotypes that are conferred by LAIV mutations in the viral RNA polymerase, while also providing insight as to why LAIVs elicit robust immune responses, despite their poor replication at human core body temperature.

## 2. Materials and Methods

Expression and Purification of Influenza A Virus RNA Polymerase Complex. Influenza A virus RNA polymerase complexes were produced via a baculovirus expression system in insect cells, as described [16]. Briefly, the PA, PB1 and PB2 genes were amplified by reverse transcription PCR from influenza strain PR8 H1N1. The C terminus of the resulting PA gene was then tagged with the tandem affinity purification (TAP) tag, consisting of a thrombin cleavage site followed by a His_6_ epitope tag, a tobacco etch virus (TEV) cleavage site, and an IgG binding domain. The polymerase mutations from the Ann Arbor (AA) and Leningrad (Len) LAIV viruses (AA = PB1: K391E, E581G, A661T; PB2: N265S; Len = PB1: K265N, V591I; PB2: V478L) were introduced into the PB1 and PB2 genes via site-directed mutagenesis, and the resulting mutant genes were cloned into the baculovirus transfer vector pVL1392 (Invitrogen, Carlsbad, CA, USA) between the BglII and Xbal restriction sites. Each plasmid was separately transfected into SF9 insect cells in ESF921 media (Expression Systems), to obtain corresponding recombinant baculoviruses encoding the proteins of interest. Virus stocks were subsequently stored at 4 °C until needed. Tni insect cells were then co-infected with appropriate combinations of recombinant viruses to allow the purification of WT or mutant/LAIV (AA/Len) influenza polymerase complexes (PA, PB1, PB2), using the TAP tag introduced into the C-terminus of PA [16].

Template Preparation. 5′ vRNA (5′-AGUAGAAACAAGGCC-3′) and the 30-nt template (5′-GCAUUGUCGCAAUCAGUACCUGCUUUCGCU-3′) which encodes for the first 30 nucleotides from the 3′ end of the PR8 PA gene were synthesized by Integrated DNA Technologies, Coralville, IA, USA). Separately, a non-specific 20-nt primer was also synthesized by the same manufacturer, for use as a loading control in transcription assays (see below).

Dinucleotide (ApG) Primed RNA Transcription. ApG primed RNA transcription assays were conducted as published [16]. Briefly, the reactions were performed in a volume of 20 µL, consisting of transcription buffer, 500 µM nonradioactive NTPs, 0.5 µM radioactive [α-32P] GTP (3000 Ci mmol-1), 5′ and 3′ vRNAs at 0.16 µM each, 0.3 mM ApG (Jena Bioscience, Jena, Germany), 0.25 U/µL RNase inhibitor (New England Biolabs, Beverly, MA, USA) and respective polymerase complexes. The transcription of a 30-nt RNA product by each purified polymerase was allowed to occur at 30 °C, 32 °C, 37 °C, 39 °C and 42 °C, for 30 min, before 10 µL of stop buffer (10 mM EDTA, pH 8.0, 90% formamide) was added to terminate the reactions. The resulting RNA products were separated by 20% PAGE in 7 M urea and visualized using an AmershamTM TyphoonTM Biomolecular Imager (Cytiva, Marlborough, MA, USA). Densitometry analyses were performed using ImageQuant TL 8.1 (Cytiva).

Cell Culture and Viral infections. Madin-Darby Canine Kidney (MDCK) cells were grown in Dulbecco’s modified Eagle medium (DMEM, Gibco, Waltham, MA, USA) supplemented with 10% fetal bovine serum (FBS, Atlanta Biologicals, Flowery Branch, GA, USA) and 1% penicillin (100 U/mL)-streptomycin (100 µg/mL) at 37 °C in air enriched with 5% CO_2_. After influenza infections, cells were switched to post-infection (P.I.) media (DMEM) supplemented with 0.3% bovine serum albumin (Sigma, St. Louis, MO, USA), 1% penicillin-streptomycin (see above) and TPCK (tolylsulfonyl phenylalanyl chloromethyl ketone) treated trypsin (2 µg/mL; Sigma). The recombinant A/Puerto Rico/8/1934 (H1N1) containing the polymerase mutations from the Ann Arbor (AA) and Leningrad (Len) LAIV viruses were generated using previously described influenza reverse genetics techniques [17]. All viral rescues and titrations involving both WT and mutated/LAIV strains were conducted at 33 °C and 37 °C. The recombinant A/Ann Arbor/6/1960 virus was generated in ten-day-old embryonated chicken eggs (Charles River labs, Wilmington, MA, USA) using previously described influenza reverse genetics techniques [1]. Viruses using the A/Ann Arbor/6/1960 genetic background (AA WT and AA LAIV) were rescued using plasmids encoding the six internal genes of AA and the HA/NA genes of A/California/07 2009 (Cal/09) (H1N1). AA LAIV contains PB1 and PB2 mutations from the A/Ann Arbor/6/1960 vaccine strain.

Real-Time PCR. To determine the levels of viral RNA species in vivo, triplicate wells of MDCK cells were infected with viruses of interest at a MOI of 1. After 1 h at room temperature, the cells were switched to P.I. media and incubated at 33 °C or 37 °C. Cells were harvested at 2, 4, 8, 12, and 24 h post-infection for RNA isolation and cell supernatants were frozen for TCID50 analysis. In vitro synthesis of RNA standards and hot-start reverse transcription was performed as described [18]. Briefly, cDNAs complementary to the three types of influenza RNA (vRNA, cRNA and mRNA) were synthesized with primers containing a unique tag unrelated to the influenza virus or mammalian cells (Table 1). Real-time PCR was performed with SYBR green qPCR Supermix on a CFX Connect Real time PCR system (BioRad. Hercules, CA, USA) with cycle conditions of 95 °C for 10 min, followed by 40 cycles of 95 °C for 15 sec and 60 °C for 1 min. All primers used for reverse transcription and qPCR are listed in Table 1. Ten-fold serial dilutions of synthetic viral RNA standards were used to generate a standard curve.

Infectious Virus Titration (TCID_50_). Supernatants from infected cell cultures and were subjected to Tissue Culture Infectious Dose assay (TCID_50_/mL) as described [19]. Mean values and standard deviations were calculated using Prism (version 10.4.1) software.

Enzyme Linked Immunosorbent assay (ELISA) for quantitation of viral proteins. Sandwich ELISAs were performed to quantitate viral protein concentrations in supernatants from virus-infected cell cultures. Briefly, for quantitation of viral nucleoprotein (NP) and non-structural protein 1 (NS1), 96 well ELISA microplates were coated with 1 µL/mL anti-NP capture antibody (Sino Biological, Houston, TX, USA, 40205-MM16) or NS1 capture antibody (Santa Cruz Biotechnology, Dallas, TX, USA NS1-23-1) and incubated overnight. Recombinant NP/NS1 protein standards (Sino Biological, 11675-V08B and 40011-V07E) and diluted cell lysate were added for 1 h. After washing (PBS + Tween 0.05%), 200 ng/mL anti-NP/anti-NS1 detection antibody (Sino Biological, 40208-R014 and Thermofisher (Waltham, MA, USA, PA5-32243) was added for 2 h followed by an HRP-conjugated secondary antibody (Biorad, Hercules, CA, USA, 1706515) for 1 h. Following the addition of TMB substrate (Biorad, 1721068), plates were read at 450 nm on a Spectramax Mini microplate reader (Molecular Devices, San Jose, CA, USA).

## 3. Results

**Figure 1:**
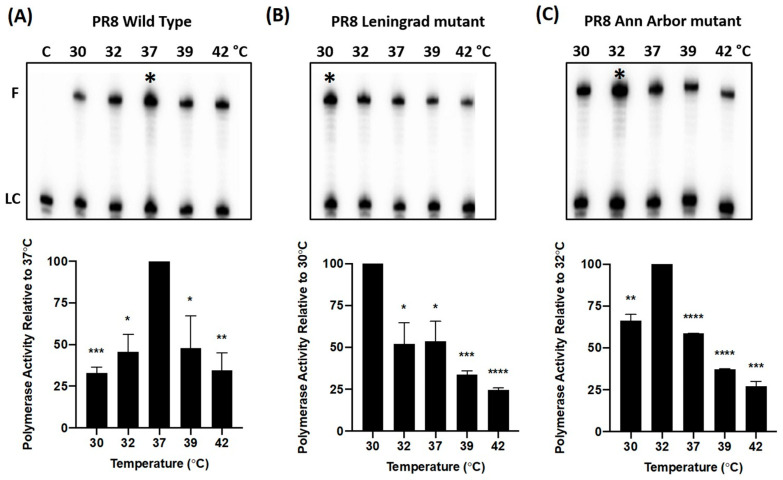
Temperature-dependent RNA synthesis profiles of purified WT and mutant PR8 influenza A virus RNA polymerase complexes. The heterotrimeric PR8 influenza virus polymerase was purified from insect cells that were co-infected with baculoviruses encoding the viral PA, PB1 and PB2 proteins (as described [16]). (Upper Panels) RNA synthesis reactions were performed using the purified polymerase complexes for 30 min at physiologically relevant temperatures (30 °C, 32 °C, 37 °C, 39 °C, 42 °C), using an ApG-primed 30-nt template corresponding to the 3′ end of the viral PA gene16. The resulting 30-nt fully extended RNA products, F, generated by the (**A**) PR8 WT, (**B**) PR8 Leningrad mutant, and (**C**) PR8 Ann Arbor mutant polymerases were visualized using a phosphorimager. Maximal polymerase activity is denoted in each panel by “*” (**A**–**C**). (Lower Panels). To quantify RNA synthetic activity, band intensities were measured and normalized to the intensity of a ^32^P-labeled 20-mer primer which was used as a loading control, LC. For each of these three experiments, polymerase activity is presented as the % of the maximal RNA synthesis efficiency (100%) across the temperature range analyzed. Data in the Lower Panel represent mean values of three independent measurements; the error bars denote the standard error of these mean values. An unpaired t test was performed to deter-mine the statistical significance of each reading (*: *p* < 0.05; **: *p* < 0.01; ***: *p* < 0.001; ****: *p* < 0.0001).

LAIV mutations in the PB1 and PB2 subunits of influenza A virus RNA polymerase complex alter its temperature-dependent transcription activity. We utilized a biochemical approach to investigate the effect(s) of the polymerase mutations from cold-adapted LAIVs (Leningrad or Ann Arbor) on the transcriptional activity of the viral polymerase complex over a range of biologically relevant temperatures (30–42 °C). ApG-primed RNA synthesis was measured at varying temperatures (30 °C, 32 °C, 37 °C, 39 °C, 42 °C), using purified WT PR8 strain polymerase and mutant derivatives of the PR8 polymerase complex, containing the LAIV-Len and LAIV-AA mutations. The individual PR8 polymerase proteins were purified from insect cells that were co-infected with baculoviruses encoding the viral PA, PB1 and PB2 proteins (as described [16]). Polymerase assays were conducted for 30 min in the presence of 500 µM of non-radioactive NTPs and 0.5 µM of the radioactive [α-32P] GTP. At the end of each reaction, a 30-nt RNA product was generated from the template which encodes for the first 30 nucleotides from the 3′end of the PA gene (Figure 1).

As observed in Figure 1A, the WT PR8 polymerase complex generated the most prominent 30-nt full-length product (F) at 37 °C. Densitometric analysis of the band intensities showed that the WT protein exhibited its maximal polymerase activity at 37 °C (Figure 1A). The activity declined at both higher and lower temperatures (68% of maximum at 30 °C, and 66% at 42 °C, respectively). In contrast to the WT polymerase, the two LAIV polymerase complexes (Leningrad, Figure 1B and Ann Arbor, Figure 1C) demonstrated maximal activity at cold temperatures (30 °C and 32 °C, respectively), and greatly reduced activity at elevated temperatures (37 °C, 39 °C, 42 °C)—consistent with the *ca* phenotype of the original LAIV-AA and LAIV-Len viruses [20]. When the LAIV polymerase complexes were tested at higher temperatures (37 °C, 39 °C, 42 °C), weaker RNA extension activities ranging from 23 to 52% (Leningrad), and 27–55% (Ann Arbor), of respective maximum activities at 30 °C, and 32 °C, were observed.

Influenza virus mutants containing LAIV mutations in the viral polymerase complex display temperature-dependent alterations in transcriptional and replicative activity in MDCK cells. To gain further insight into the effects of the LAIV mutations on PB1 and PB2 on viral polymerase function, we infected MDCK cells with WT PR8 virus and mutated derivatives of PR8 that contain the Leningrad and Ann Arbor LAIV mutations in PB1 and PB2, and then measured the production of the three species of viral RNA: mRNA, cRNA, and vRNA, using a previously described qPCR method [18]. The NP residue at position 34 is glycine in the PR8 backbone used. This substitution is also present in the Ann Arbor LAIV strains, where it has been implicated—together with other polymerase mutations—in contributing to temperature-sensitive and attenuated phenotypes [14]. Because glycine was already present at this position in both our PR8 and AA-based viruses, NP 34G was not a variable in our experiments and was therefore not analyzed separately.

**Figure 2:**
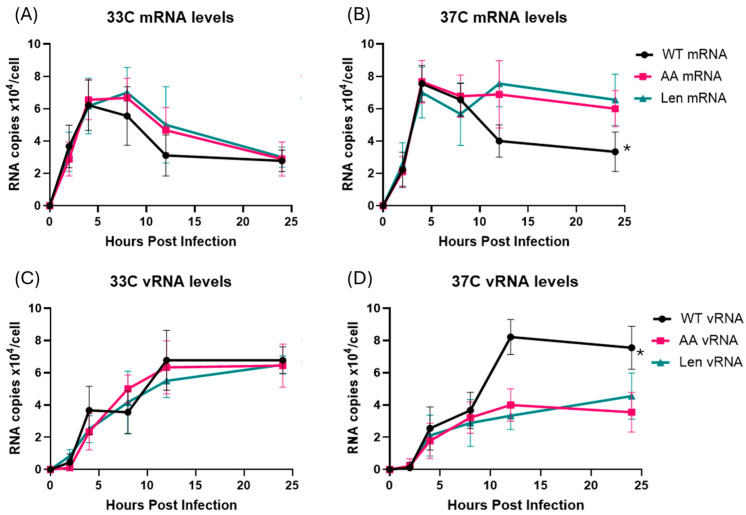
Production of viral mRNA and genomic vRNA in MDCK cells infected with WT and mutant PR8 influenza A viruses at 33 °C and 37 °C. MDCK cells were infected with WT PR8 virus or the indicated LAIV mutant strains (PR8-AA and PR8-Len) at 33 °C or 37 °C, at an MOI of 1 and harvested at 2, 4, 8, 12 and 24 h post-infection. Viral mRNA and genomic vRNA species were quantified by qPCR using specific primers and a synthetic RNA standard curve (see Methods). (**A**,**C**) Viral mRNA and genomic vRNA levels in MDCK cells infected by the indicated viruses at 33 °C. (**B**,**D**) Viral mRNA and genomic vRNA levels in MDCK cells infected by the indicated viruses at 37 °C. Data represent mean values of three independent measurements; the error bars denote the standard error of these mean values. Area under the curve analysis was performed using RNA measurements taken at multiple timepoints using the trapezoidal method in GraphPad Prism (v10.4.1). A mixed-effects model was used to account for repeated measures and potential missing values across replicates. *: *p* < 0.05.

Briefly, we designed cDNA synthesis primers based on the PR8 PA gene and added specific tags that discriminate between the 3 different RNA species in each sample (Table 1). We then infected MDCK cells with WT PR8, PR8-Len and PR8-AA at 33 °C and 37 °C, at an MOI of 1, and harvested the cells at 2, 4, 8, 12 and 24 h post-infection for subsequent isolation of RNA (Figure 2).

**Figure 3:**
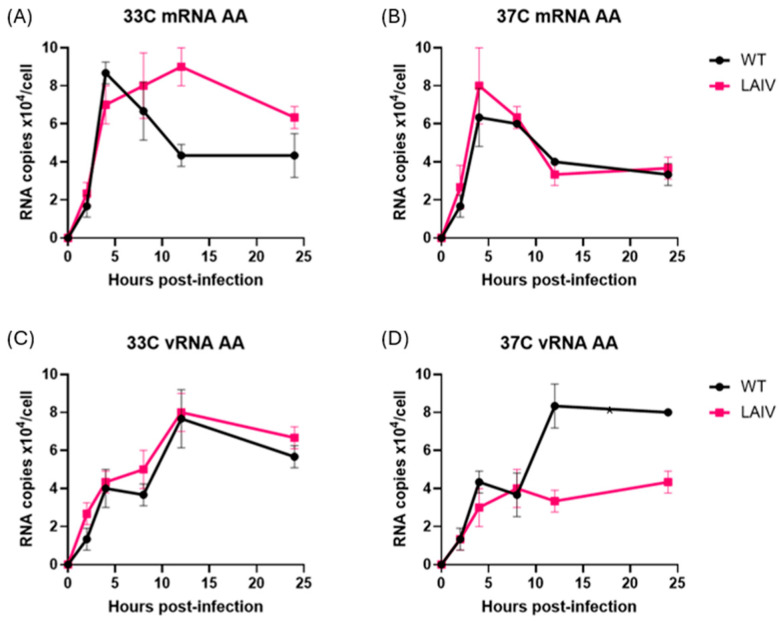
Time course of production of vRNA and mRNA species in MDCK cells infected with AA-WT, AA-LAIV viruses at 33 °C and 37 °C. MDCK cells were infected with A/Ann Arbor/6/1960 (WT and LAIV) at an MOI of 1, incubated at 33 °C (**A**,**B**) or 37 °C (**C**,**D**), and then harvested at 2, 4, 8, 12, and 24 h post-infection. To differentiate each species of RNA (vRNA, cRNA and mRNA), unique tags were assigned to each cDNA synthesis primer to be used in subsequent qPCR for detection. (**A**,**B**) RNA levels of indicated Influenza strains incubated at 33 °C. (**C**,**D**) RNA levels of indicated Influenza strains incubated at 37 °C. Data represent mean values of three independent measurements; the error bars denote the standard error of these mean values. Area under the curve analysis was performed using RNA measurements taken at multiple timepoints using the trapezoidal method in GraphPad Prism (v10.4.1). A mixed-effects model was used to account for repeated measures and potential missing values across replicates.

Infection of MDCK cells at 33 °C by both WT PR8 and LAIV PR8 viruses (PR8-Len, PR8-AA) strains resulted in comparable production of mRNA and vRNA by each of the viruses, at all timepoints examined (Figure 2A,C). Viral mRNA levels peaked early, and declined over time, while vRNA levels increased gradually over 24 h, consistent with previous findings from other groups [21]. In contrast, infection of MDCK at 37 °C by WT PR8 and LAIV PR8 viruses yielded divergent results. Specifically, the two LAIV PR8 viruses (PR8-Len and PR8-AA) produced approximately half as much vRNA at 12 and 24 h post-infection and twice the amount of mRNA, compared to WT PR8 (Figure 2B,D). Additionally, we performed a similar infection experiment in MDCK cells, with WT PR8 and PR8-AA at an MOI of 0.1 (Supplementary Figure S2). As expected, the time required to reach peak levels of mRNA and vRNA production was increased at both 33 °C and 37 °C (Supplementary Figure S2), as compared to cells infected at the higher MOI (Figure 2). However, just as was the case in cells infected with LAIV PR8 viruses at an MOI of 1, cells infected with PR8 AA at 37 °C, an MOI of 0.1 contained strikingly reduced levels of vRNA (by approximately 3-fold) at 24 h post-infection (Supplementary Figure S2D). This finding suggests that the observed defect in vRNA production by LAIV PR8 viruses in MDCK cells at 37 °C is independent of the MOI used to establish viral infection. To confirm that this observation was not unique to the viral genetic background of PR8, we repeated this experiment using a recombinant A/Ann Arbor/6/1960 virus strain containing the Cal/09 HA/NA genes—along with a mutant version of the same virus containing the LAIV polymerase mutations from *ca* AA (i.e., PB1: K391E, E581G, A661T; PB2: N265S). Data from this experiment revealed similar results to those observed with the WT PR8 and LAIV PR8 viruses (Figure 3). Specifically, the LAIV AA virus produced approximately half as much vRNA at 12 and 24 h post-infection and similar levels of mRNA, compared to WT AA (Figure 3).

Collectively, these virus infection experiments with matched WT and LAIV mutant strains show that polymerase mutations present in ca LAIVs confer a selective, temperature-dependent defect on the replicase activity of the viral RNA polymerase, that may be secondary to a failure to transition from viral transcription to replication.

**Figure 4:**
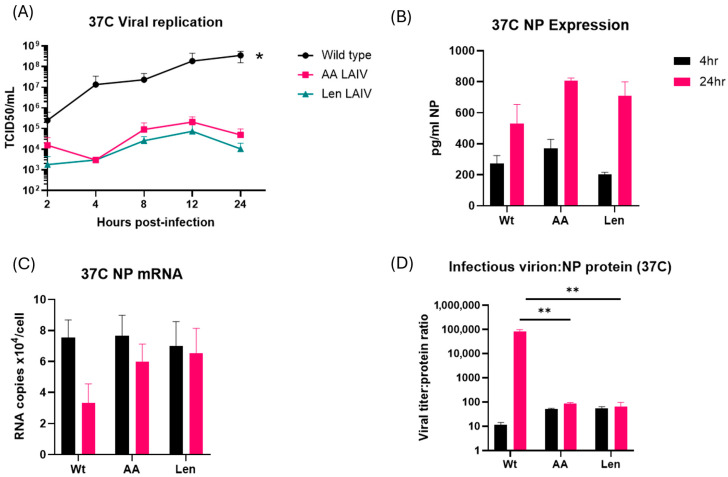
Comparative analysis of viral replication, protein production and mRNA levels in MDCK cells infected with WT and mutant PR8 influenza A viruses at 37 °C. MDCK cells were infected with WT PR8 virus or the indicated LAIV mutant strains (PR8-AA and PR8-Len) at 37 °C, at an MOI of 1 and harvested at the indicated timepoints thereafter (up to a maximum of 24 h post-infection). (**A**) Levels of infectious virus progeny in supernatants from MDCK cells infected with the indicated viruses were quantified by TCID_50_ assay at 2, 4, 8, 12 and 24 h post-infection. Area under the curve analysis was performed using viral titer measurements taken at multiple timepoints using the trapezoidal method in GraphPad Prism (v10.4.1). A mixed-effects model was used to account for repeated measures and potential missing values across replicates. (*: *p* < 0.05). (**B**) Levels of viral NP in lysates from MDCK cells infected with the indicated viruses were quantified by ELISA at 4 and 24 h post-infection. An unpaired t test was performed to determine the statistical significance of each reading (**C**) Levels of viral NP mRNA in lysates from MDCK cells infected with the indicated viruses were quantified by qPCR assay at 4 and 24 h post-infection. An unpaired t test was performed to determine the statistical significance of each reading (**D**) The relative ratio of infectious virus production to levels of intracellular NP is presented for MDCK cells infected with the indicated viruses at 4 and 24 h post-infection. An unpaired t test was performed to determine the statistical significance of each reading (**: *p* < 0.01. Data represent mean values of three independent measurements; the error bars denote the standard error of these mean values.

Influenza virus containing LAIV mutations in the viral polymerase complex produce similar levels of viral proteins at 37 °C, but greatly reduced levels of infectious viral progeny. We next tested whether the temperature-dependent changes in viral RNA synthesis by LAIV strains result in changes in viral protein synthesis and/or production of infectious viral progeny. MDCK cells were infected with WT PR8 and LAIV PR8 (PR8-Len, PR8-AA) viruses at 37 °C at an MOI of 1.0, and culture supernatants were harvested at selected timepoints over a 24 h time period. TCID_50_ analysis showed that WT PR8 generated between 2 log_10_ and 3 log_10_ higher levels of infectious virus progeny at 24 h, compared, respectively, to PR8-AA and PR8-Len (Figure 4A). In contrast, the levels of viral NP production by the various viruses were very similar at both 4 and 24 h post-infection (see Figure 4B; the PR8-AA and PR8-Len viruses produced slightly more NP protein than WT PR8, but this difference was not statistically significant). Consistent with this, the levels of NP mRNA were also very similar in cells infected by both WT PR8 and LAIV PR8 (Figure 4C). Perhaps most strikingly, and as would be expected given the collective findings in Figure 4A–C, the ratio of infectious virion production to viral protein production was significantly (3 log_10_) higher for WT PR8 at 24 h post-infection of MDCK cells at 37 °C, compared to that for PR8-Len and PR8-AA (Figure 4D). Finally, we tested whether our findings in terms of viral NP production could be confirmed with a second viral protein. For this purpose, we chose to focus on the viral NS1 protein—and found similar levels of NS1 production (Supplementary Figure S4) by the WT and LAIV PR8 strains, though there was a decrease in NS1 levels in LAIV PR8 strains at 6 h post-infection (results non-significant). Collectively, these data show that although the PR8-AA and PR8-Len strains are able to produce mRNA and protein at comparable levels to WT PR8 virus, they produce much lower levels of infectious virions at higher temperatures.

## 4. Discussion

Previous studies by our lab and others have shown that mutations in the viral RNA polymerase PB1 and PB2 subunits found in the LAIV strains, A/Ann Arbor/6/1960 H2N2 and A/Leningrad/17/57 H2N2, are necessary and sufficient to confer temperature sensitive (ts) and cold-adapted (ca) phenotypes on other strains of influenza A viruses, including PR8 (H1N1) [13,15]. However, the mechanistic basis for this is not well understood. In this study, we therefore set out to address this knowledge gap. Using a cell-free assay system, we examined the temperature sensitivity of purified PR8 RNA polymerase complexes containing mutations present in LAIV-AA or LAIV-Len. These studies revealed that the LAIV polymerases exhibited maximal transcriptional activity at lower temperatures (30–32 °C) than the WT PR8 RNA polymerase complex (37 °C), consistent with the ts and ca phenotypes of the corresponding LAIV viruses [13]. We next examined polymerase activity in cell-based systems, using infectious influenza viruses. In designing these experiments, we considered a previously reported finding that a mutated derivative of the A/California/10/78 (H1N1) virus containing the AA LAIV mutations produced much lower levels of infectious virus progeny in primary human nasal epithelial cells, but far higher levels of viral mRNA, than its WT counterpart [21]. We therefore hypothesized that LAIV mutations might exert differential effects on viral mRNA transcription versus viral genome replication. To test this theory, we measured viral mRNA and viral genomic RNA levels in influenza-infected MDCK cells, using a previously characterized qPCR method [18] to quantitatively measure viral mRNA as well as genomic vRNA and cRNA. These experiments revealed that, at 37 °C (but not at lower temperatures), PR8 viruses containing the PB1 and PB2 mutations from LAIV-Len and LAIV-AA produced elevated levels of viral mRNA and reduced levels of genomic vRNA when compared to WT PR8. Very similar findings were also obtained when these same mutations ere introduced into the genetic background of the recombinant A/Ann Arbor/6/1960 virus. In addition, cRNA measurements showed that cRNA levels were not substantially reduced, suggesting that decreased vRNA synthesis is not simply due to impaired cRNA production (Figure S1).

It is important to note that the relative abundance of viral mRNA versus genomic RNA can also be influenced by the multiplicity of infection (MOI), as different MOIs can lead to distinct replication kinetics and alter the timing of transcription and replication phases [22]. We therefore performed infection experiments at both high (1.0) (Figure 2) and low (0.1) (Figure S2) MOIs—and observed a similar, selective impairment of vRNA synthesis in cells infected by PR8 LAIV strains at 37 °C (but not at 33 °C).

In addition to these differential changes in viral RNA production, our studies also show a profound (2–3 log_10_) decrease in the production of mature infectious virions in MDCK cells by PR8-LAIV strains (PR8-Len, PR8-AA) when compared to WT PR8. Consistent with this, the ratio of either viral mRNA or viral protein levels to infectious progeny virus titers was much (2–3 log_10_) higher in MDCK cells infected with the PR8-LAIV viruses than PR8-WT, at 37 °C. This, in turn, concords with the profound attenuation but robust antigen production (and resultant immunogenicity) of LAIVs, at elevated temperatures—when compared to WT influenza virus [7,23,24]. As noted above, the changes in viral RNA synthesis, and the failure of LAIVs to fully transition from viral mRNA synthesis to the production of genomic vRNA may reflect temperature-dependent changes in the conformation of the viral polymerase complex (e.g., from a transcriptional complex to a replicase complex) [25,26,27,28]. In addition, the interaction of the polymerase complex with cellular proteins such as ANP32A or the viral protein NS2 [25,26,27,28] may also influence the propensity of the tripartite polymerase complex to undergo changes in structure, including the adoption of an encapsidase-competent conformation [28]. This may in part explain the discordance between the very large effect of the LAIV mutations on the production of infectious viral progeny (2–3 log_10_) and their much more modest effect on viral RNA synthesis (approx 2-fold) [28].

One limitation of our cell culture experiments in that they were performed exclusively in MDCK cells. These cells have some significant differences from primary human airway epithelial cells—including the fact that, while interferon-competent, MCDK cells have “*an insufficient interferon-stimulated antiviral state*” [29]. This may contribute to the greater degree of attenuation of LAIV replication that has been observed by other investigators in primary human airway epithelial cells, as compared to MDCK cells [8,30]. Thus, future studies in additional cell types, including primary human airway epithelial cells may be important to understanding the full significance of the findings reported in this manuscript.

**Figure 5:**
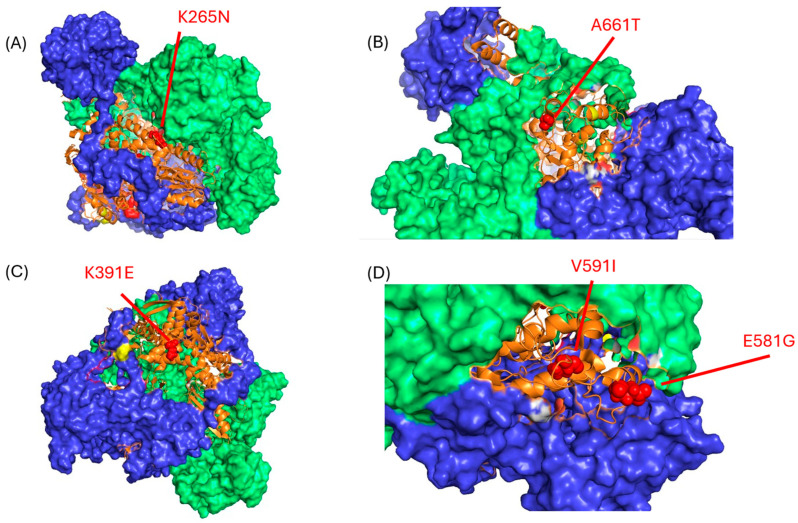
LAIV mutation locations in the PB1 subunit of the influenza A virus polymerase complex. The locations of the PB1 mutations found in AA and Len LAIV strains are shown as red spheres ((**A**): K265N; (**B**): A661T; (**C**): K391E; (**D**): V591I and E581G) on the background of the WT influenza A/Northern Territory/60/1968 H3N2 polymerase complex (PDB 6rr7); mutations K265N and V591I derive from LAIV-Len, while mutations K391E, E581G and A661T derive from LAIV-AA. The orange ribbon structures indicate PB1, while the blue and green surface structures represent PA and PB2, respectively. Notably, the majority of the LAIV mutations map to interface domains between subunits of the heterotrimeric IAV polymerase complex. Images were generated using Pymol (version 3.0.1) software.

Finally, the crystal structures of H1N1 RNA polymerases reveal that many of the LAIV PB1 and PB2 mutations lie along the interfaces between polymerase subunits [14], including A661T, which lies in a region where PB1 and PB2 interact and along the PB1:RNA interface (Figure 5). We hypothesize that this may impact the physical/conformational stability of the purified “PR8-LAIV” RNA polymerase complexes in a temperature-dependent manner and thereby (for example) regulate the balance between viral replicase activity and mRNA transcriptional activity [25,26,27,28]. We also mapped PB1 and PB2 LAIV mutations to the crystal structures of the asymmetric polymerase dimer bound to ANP32A (PDB:8RMR) but found that the mutated residues are not close to interfaces between ANP32A and polymerase components, or to interfaces between the polymerase dimers. We therefore speculate that the LAIV mutations may confer more subtle, temperature-sensitive, structural changes on the conformation (and associated functional activity) of the influenza replication complex, including (potentially) the regulation of the transcription-to-replication switch. Further experiments will be needed to evaluate this hypothesis.

In summary, our data show that polymerase mutations present in LAIV strains alter the balance of viral Mrna mRNA vs. viral genomic RNA production at elevated temperatures, while also resulting in high levels of viral protein production but drastically reduced yields of infectious virus progeny. In many respects, these are ideal characteristics for an attenuated virus vaccine strain. Our findings also suggest that it may be possible to achieve even greater attenuation of progeny virus titers, along with higher levels of viral antigen production—and thus, improved new LAIV strains—through new combinations of viral polymerase mutations.

## Figures and Tables

**Table 1:** Primer sequences used for strand-specific quantification of influenza A virus NP RNA species. Strand-specific primer sets used in quantitative RT-PCR assays to detect influenza A nucleoprotein (NP) mRNA, complementary RNA (cRNA), and viral RNA (vRNA). Separate primer sets were designed for the NP gene of the A/Puerto Rico/8/1934 strain (PR8 NP) and the A/Ann Arbor/6/1960 strain (AA NP) to account for sequence differences between viral backbones. Forward and reverse primers were designed to selectively amplify each RNA species based on unique sequence features. Primer sequences are listed in the 5′ to 3′ direction.

Target Gene	Purpose	Primer Name	Sequence (5′-3′)
PR8 NP vRNA	Reverse transcription	vRNAtag_PR8NP_348F	GGCCGTCATGGTGGCGAATCGGAAAGTGGATGAGAGAACTCATC
	Real-time PCR	vRNAtag_PR8NP	GGCCGTCATGGTGGCGAAT
		PR8NP_489R	CGAACAAGAGCTCTTGTCCTCTGA
PR8 NP cRNA	Reverse transcription	cRNAtag_PR8NP_1565R	GCTAGCTTCAGCTAGGCATCAGTAGAAACAAGGGTATTTTTCTTT
	Real-time PCR	cRNAtag_PR8NP	GCTAGCTTCAGCTAGGCATC
		PR8NP_1467F	GATCGTGCCTTCCTTTGAC
PR8 NP mRNA	Reverse transcription	mRNAtag_PR8NP_dTR	CCAGATCGTTCGAGTCGTTTTTTTTTTTTTTTTTCTTTAATTATCG
	Real-time PCR	mRNAtag_PR8NP	CCAGATCGTTCGAGTCGT
		PR8NP_1467F	GATCGTGCCTTCCTTTGAC
AA NP vRNA	Reverse transcription	vRNAtag_AANP_264F	GGCCGTCATGGTGGCGAATGAGGAGGAATAAATATCTGGAAGAA
	Real-time PCR	vRNAtag_AANP	GGCCGTCATGGTGGCGAAT
		AANP_390R	TTGGCGCCAGATTCGCCTTATT
AA NP cRNA	Reverse transcription	cRNAtag_AANP_1566R	GCTAGCTTCAGCTAGGCATCAGTAGAAACAAGGGTAATTTTTCCTT
	Real-time PCR	cRNAtag_AANP	GCTAGCTTCAGCTAGGCATC
		AANP_1476F	CTCTTTTGACATGAGTAATGAAG
AA NP mRNA	Reverse transcription	mRNAtag_AANP_dTR	CCAGATCGTTCGAGTeeCGTTTTTTTTTTTTTTTTTCCTTAATTGTCG
	Real-time PCR	mRNAtag_AANP	CCAGATCGTTCGAGTCGT
		AANP_1476F	CTCTTTTGACATGAGTAATGAAG

## Data Availability

The original contributions presented in this study are included in the article/Supplementary Materials. Further inquiries can be directed to the corresponding authors.

## Supplementary Materials

The following supporting information can be downloaded at https://www.mdpi.com/article/10.3390/v17111412/s1, Figure S1: Time course of production of vRNA, cRNA and mRNA species in MDCK cells infected with PR8-WT, PR8-AA and PR8-Len viruses at 33 °C and 37 °C at an MOI of 1; Figure S2: Time course of production of vRNA and mRNA species in MDCK cells infected with PR8-WT and PR8-AA viruses at 33 °C and 37 °C at an MOI of 0.1; Figure S3: Comparative analysis of viral replication, protein production and mRNA levels in MDCK cells infected with WT and mutant PR8 influenza A viruses at 33 °C; Figure S4: Measurement of influenza NS1 protein in infected MDCK cells at 33 °C and 37 °C.

## Abbreviations

The following abbreviations are used in this manuscript:

LAIV	Live Attenuated Influenza Vaccine
PR8	A/Puerto Rico/8/1934
AA	A/Ann Arbor/6/1960
Len	A/Leningrad/134/17/1957
