# Supplementary material for: LAIV Mutations Selectively Alter Influenza Viral RNA Polymerase Function, Favoring Transcription over Genome Synthesis
Source: Viruses. 2025 Oct 23;17(11):1412. doi: 10.3390/v17111412 (PMC12656728; doi:10.3390/v17111412)
Supplement: Supplementary file 1 [file viruses-17-01412-s001.zip › Justin Manuscript Supp figures Viruses 101325.pptx]

## Slide 1
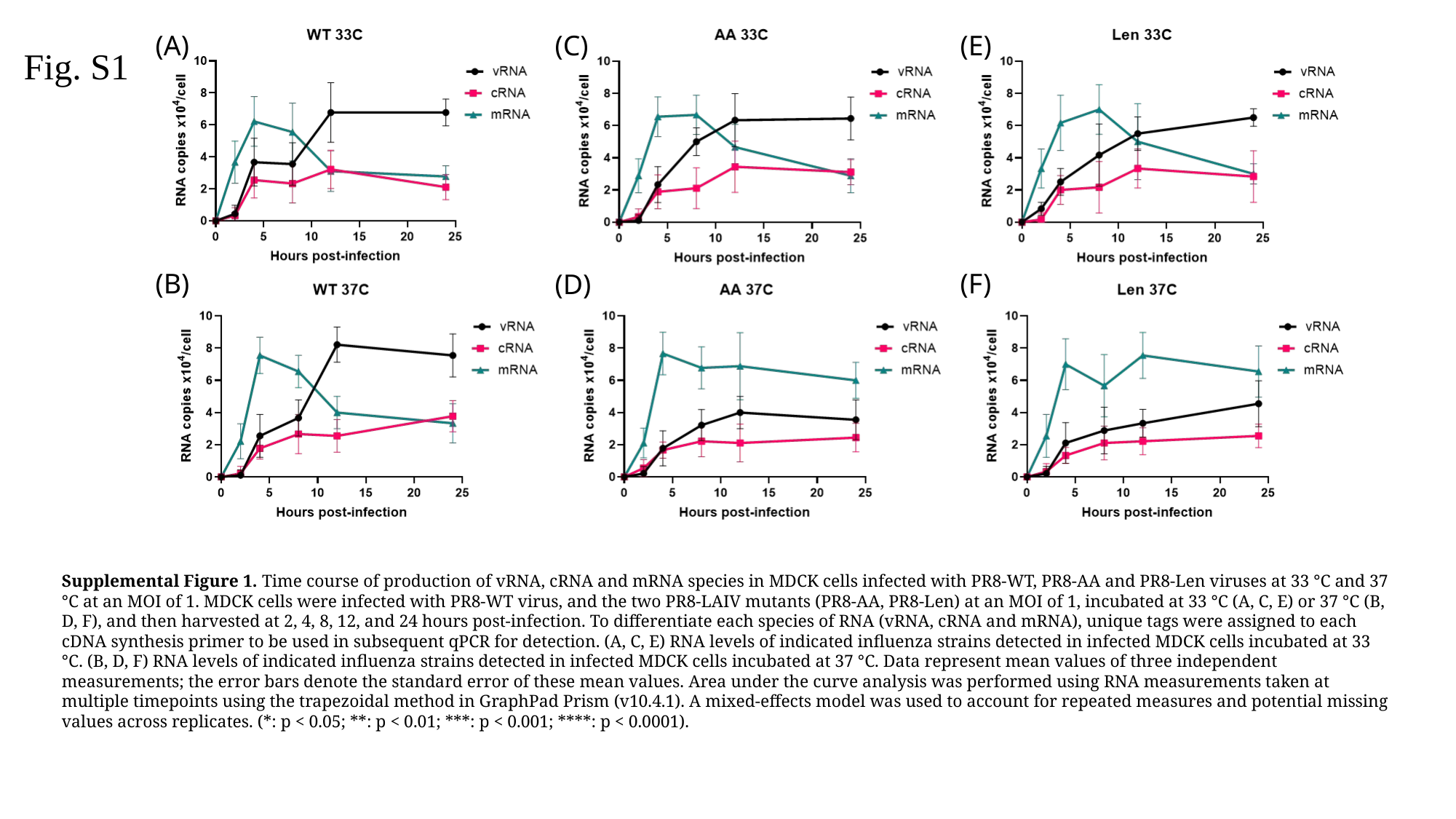

# Fig. S1
(A)
(C)
(E)
(B)
(F)
(D)
Supplemental Figure 1. Time course of production of vRNA, cRNA and mRNA species in MDCK cells infected with PR8-WT, PR8-AA and PR8-Len viruses at 33 °C and 37 °C at an MOI of 1. MDCK cells were infected with PR8-WT virus, and the two PR8-LAIV mutants (PR8-AA, PR8-Len) at an MOI of 1, incubated at 33 °C (A, C, E) or 37 °C (B, D, F), and then harvested at 2, 4, 8, 12, and 24 hours post-infection. To differentiate each species of RNA (vRNA, cRNA and mRNA), unique tags were assigned to each cDNA synthesis primer to be used in subsequent qPCR for detection. (A, C, E) RNA levels of indicated influenza strains detected in infected MDCK cells incubated at 33 °C. (B, D, F) RNA levels of indicated influenza strains detected in infected MDCK cells incubated at 37 °C. Data represent mean values of three independent measurements; the error bars denote the standard error of these mean values. Area under the curve analysis was performed using RNA measurements taken at multiple timepoints using the trapezoidal method in GraphPad Prism (v10.4.1). A mixed-effects model was used to account for repeated measures and potential missing values across replicates. (*: p < 0.05; **: p < 0.01; ***: p < 0.001; ****: p < 0.0001).

## Slide 2
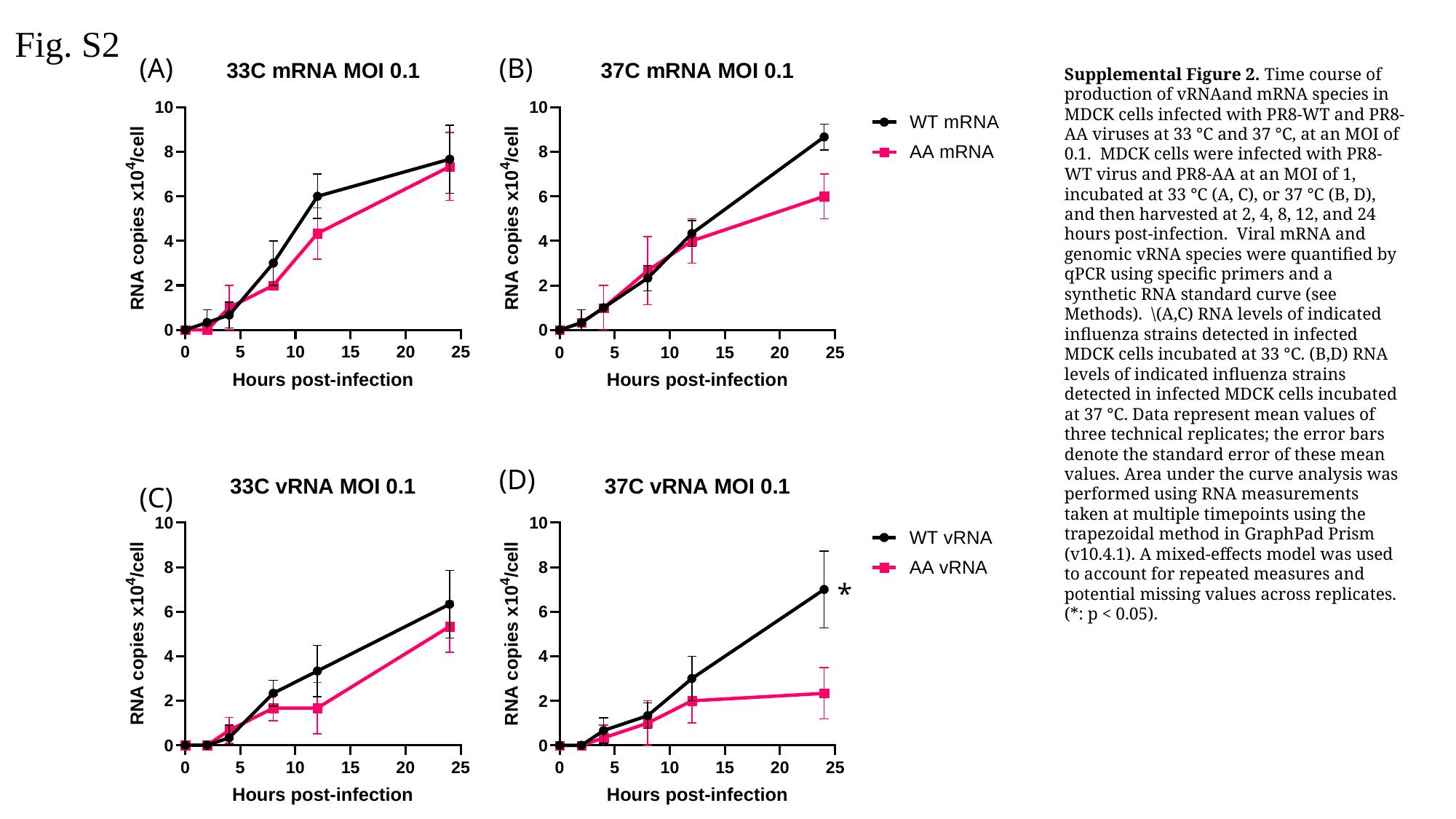

# Fig. S2
(A)
(B)
Supplemental Figure 2. Time course of production of vRNAand mRNA species in MDCK cells infected with PR8-WT and PR8-AA viruses at 33 °C and 37 °C, at an MOI of 0.1. MDCK cells were infected with PR8-WT virus and PR8-AA at an MOI of 1, incubated at 33 °C (A, C), or 37 °C (B, D), and then harvested at 2, 4, 8, 12, and 24 hours post-infection. Viral mRNA and genomic vRNA species were quantified by qPCR using specific primers and a synthetic RNA standard curve (see Methods). \(A,C) RNA levels of indicated influenza strains detected in infected MDCK cells incubated at 33 °C. (B,D) RNA levels of indicated influenza strains detected in infected MDCK cells incubated at 37 °C. Data represent mean values of three technical replicates; the error bars denote the standard error of these mean values. Area under the curve analysis was performed using RNA measurements taken at multiple timepoints using the trapezoidal method in GraphPad Prism (v10.4.1). A mixed-effects model was used to account for repeated measures and potential missing values across replicates. (*: p < 0.05).
(D)
(C)
*

## Slide 3
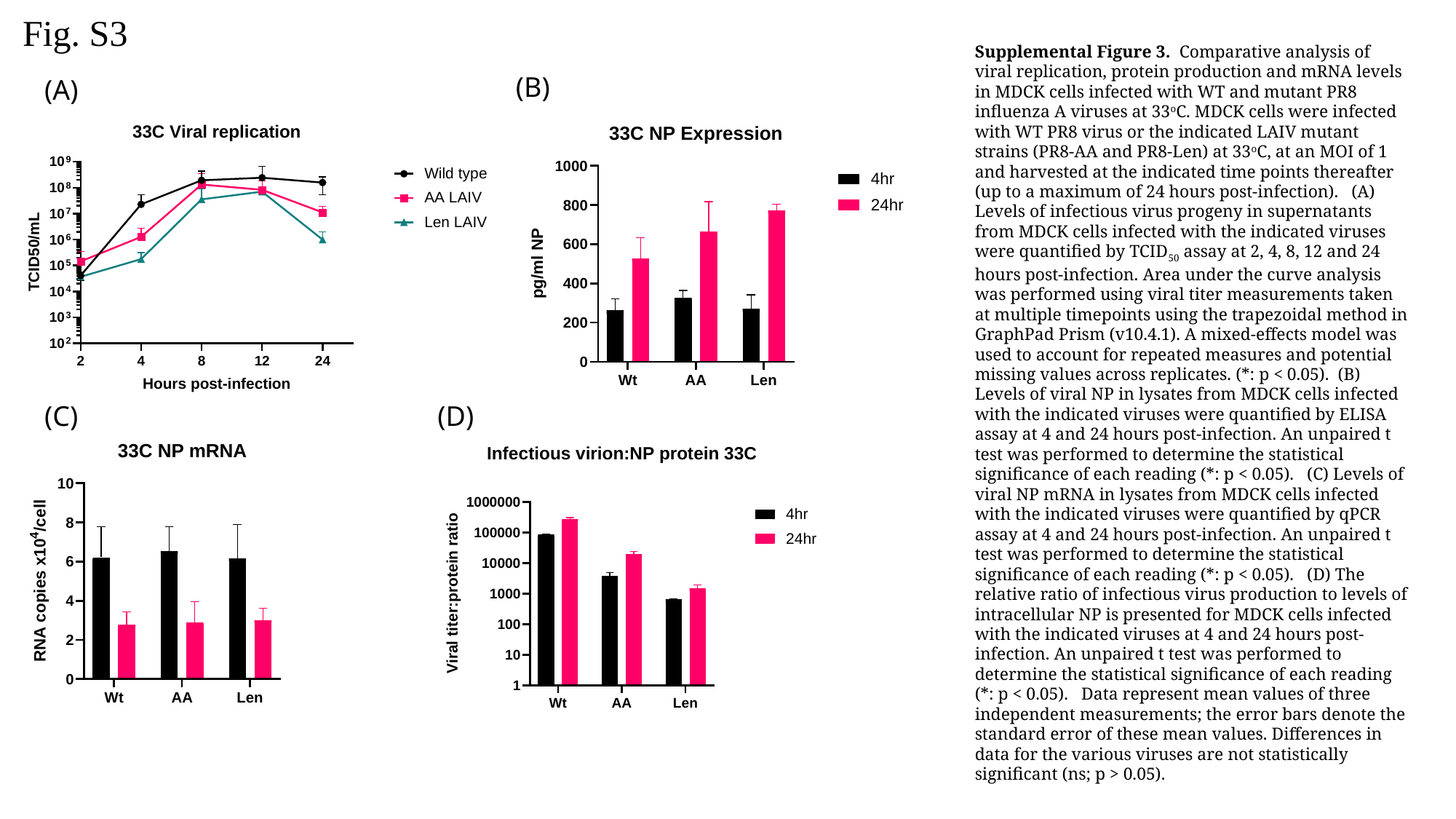

# Fig. S3
Supplemental Figure 3. Comparative analysis of viral replication, protein production and mRNA levels in MDCK cells infected with WT and mutant PR8 influenza A viruses at 33oC. MDCK cells were infected with WT PR8 virus or the indicated LAIV mutant strains (PR8-AA and PR8-Len) at 33oC, at an MOI of 1 and harvested at the indicated time points thereafter (up to a maximum of 24 hours post-infection). (A) Levels of infectious virus progeny in supernatants from MDCK cells infected with the indicated viruses were quantified by TCID50 assay at 2, 4, 8, 12 and 24 hours post-infection. Area under the curve analysis was performed using viral titer measurements taken at multiple timepoints using the trapezoidal method in GraphPad Prism (v10.4.1). A mixed-effects model was used to account for repeated measures and potential missing values across replicates. (*: p < 0.05). (B) Levels of viral NP in lysates from MDCK cells infected with the indicated viruses were quantified by ELISA assay at 4 and 24 hours post-infection. An unpaired t test was performed to determine the statistical significance of each reading (*: p < 0.05). (C) Levels of viral NP mRNA in lysates from MDCK cells infected with the indicated viruses were quantified by qPCR assay at 4 and 24 hours post-infection. An unpaired t test was performed to determine the statistical significance of each reading (*: p < 0.05). (D) The relative ratio of infectious virus production to levels of intracellular NP is presented for MDCK cells infected with the indicated viruses at 4 and 24 hours post-infection. An unpaired t test was performed to determine the statistical significance of each reading (*: p < 0.05). Data represent mean values of three independent measurements; the error bars denote the standard error of these mean values. Differences in data for the various viruses are not statistically significant (ns; p > 0.05).
(B)
(A)
(C)
(D)

## Slide 4
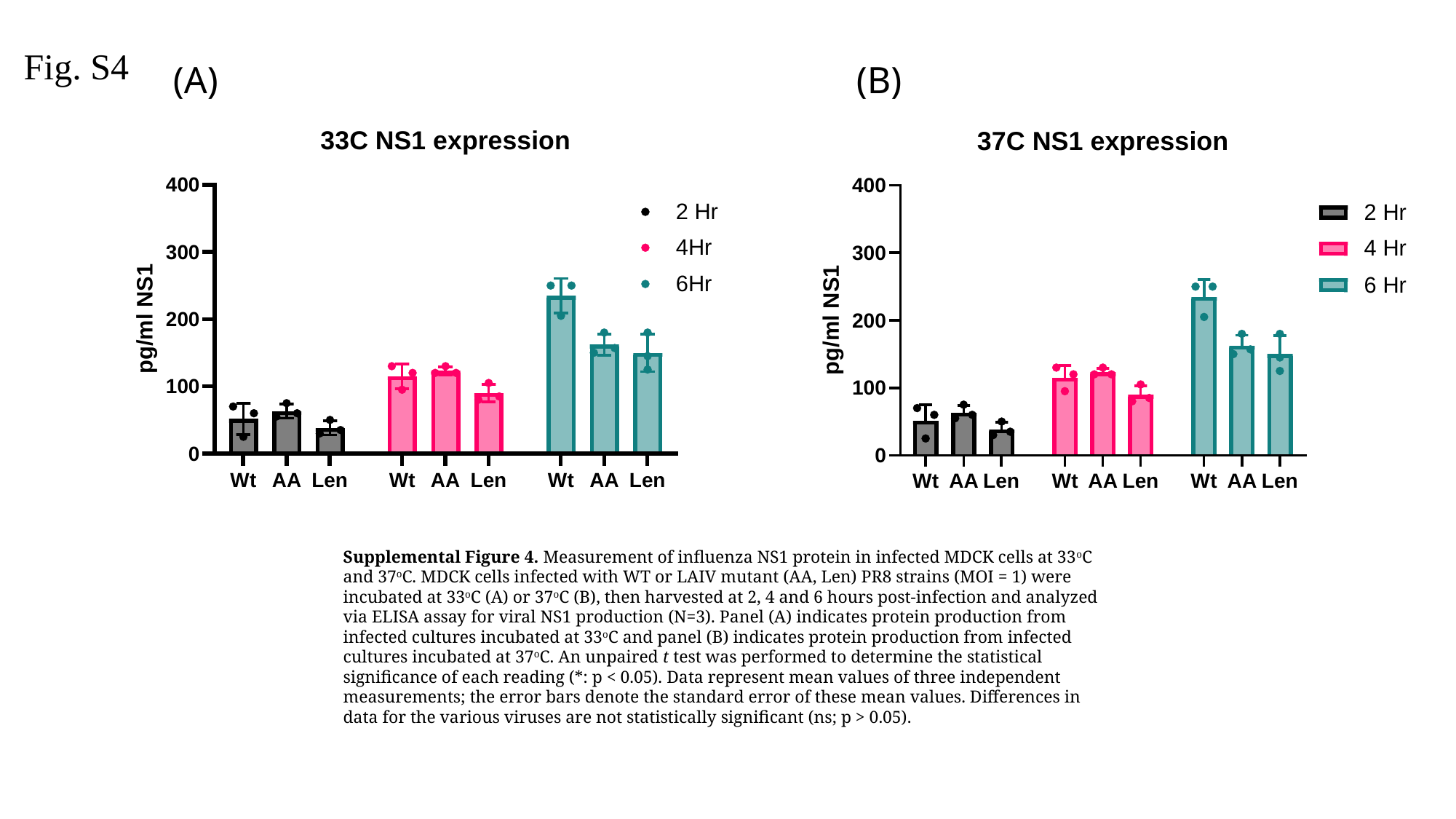

# Fig. S4
(B)
(A)
Supplemental Figure 4. Measurement of influenza NS1 protein in infected MDCK cells at 33oC and 37oC. MDCK cells infected with WT or LAIV mutant (AA, Len) PR8 strains (MOI = 1) were incubated at 33oC (A) or 37oC (B), then harvested at 2, 4 and 6 hours post-infection and analyzed via ELISA assay for viral NS1 production (N=3). Panel (A) indicates protein production from infected cultures incubated at 33oC and panel (B) indicates protein production from infected cultures incubated at 37oC. An unpaired t test was performed to determine the statistical significance of each reading (*: p < 0.05). Data represent mean values of three independent measurements; the error bars denote the standard error of these mean values. Differences in data for the various viruses are not statistically significant (ns; p > 0.05).
